# Improving Vaginal Health with a Zinc-Containing Vaginal Hydrogel

**DOI:** 10.3390/gels11030214

**Published:** 2025-03-19

**Authors:** Dávid Rátonyi, Barbara Kozma, Attila G. Sipos, Zoárd Tibor Krasznai, Bence Kozma, Peter Takacs

**Affiliations:** 1 Department of Obstetrics and Gynecology, Faculty of Medicine, University of Debrecen, Nagyerdei krt. 98., 4032 Debrecen, Hungary; ratonyi.david@med.unideb.hu (D.R.); kozma.barbara@med.unideb.hu (B.K.); sipos.attila.gergely@med.unideb.hu (A.G.S.); krasznai.zoard@med.unideb.hu (Z.T.K.); 2 Doctoral School of Nutrition and Food Sciences, University of Debrecen, Egyetem tér 1., 4032 Debrecen, Hungary; 3 Division of Female Pelvic Medicine and Reconstructive Surgery, Department of Obstetrics and Gynecology, Eastern Virginia Medical School, 825 Fairfax Avenue, Suite 526, Norfolk, VA 23507-2007, USA

**Keywords:** zinc-containing hydrogel, vulvovaginitis, vaginal health, non-hormonal treatment, local treatment

## Abstract

Vulvovaginal symptoms affect up to 39% of women. These symptoms have a significant impact on quality of life and are often linked to imbalances in the vaginal microbiota. This study evaluates the therapeutic efficacy of a zinc-containing hydroxyethyl cellulose-based hydrogel in 37 women with different vulvovaginal symptoms (itching, burning, irritation, pain, dryness, discharge, and odor). Over 12 weeks, participants applied the gel intravaginally with both assessments conducted at baseline and follow-ups. Results revealed substantial improvements in symptoms, including reductions in vaginal discharge, itching, and burning, as measured by the Vulvovaginal Symptom Questionnaire (VSQ-21), with scores decreasing from 10.78 ± 3.66 at baseline to 3.17 ± 4.16 at week 12 (*p* < 0.01). Vaginal Health Index (VHI) scores improved significantly, from 20.78 ± 1.74 at baseline to 23.64 ± 2.59 (*p* < 0.01). Cervicovaginal lavage (CVL) zinc levels decreased from 110 ± 102 µg/L at baseline to 62 ± 48 µg/L at week 4 (*p* < 0.01), increased to 80 ± 55 µg/L at week 8 (*p* = 0.04), and reached 99 ± 92 µg/L by week 12 (NS). A correlation analysis showed an inverse relationship between baseline CVL zinc levels and VSQ-21 scores (r = −0.3586, *p* = 0.034), while no significant correlation was observed with VHI scores (r = −0.0187, *p* = 0.9545). Vaginal pH levels decreased significantly, dropping from 4.03 ± 0.42 to 3.71 ± 0.48 (*p* < 0.01). These findings support the gel’s role as an effective, nonhormonal, drug-free, and local adjunct treatment for a variety of vulvovaginal symptoms.

## 1. Introduction

Vulvovaginal symptoms are one of the most commonly reported complaints in daily gynecological clinical practice [1]. They may manifest as vulvar itching, pain, skin irritation, burning sensation, vaginal discharge, unpleasant odor, dysuria, and dyspareunia, and can result from both inflammatory and non-inflammatory causes. Symptoms associated with vulvovaginitis (VV) affect approximately 15–39% of women [2,3]. The main cause of these symptoms is infection. Vulvovaginal candidiasis (VVC), trichomoniasis, and bacterial vaginosis (BV) are the most frequent causes, and these are responsible for 90% of cases in symptomatic women [1]. Non-infectious causes of vaginitis should also be considered, such as postpartum vulvovaginal atrophy in women of reproductive age or genitourinary syndrome of menopause (GSM) in postmenopausal women, which affects 50–60% of the postmenopausal population [4,5].

Chronic vulvovaginal conditions significantly impact quality of life, often leading to profound reductions in physical comfort, sexual function, and perceived overall health status. Previous studies have documented that recurrent vulvovaginal candidiasis negatively affects patients’ physical well-being, diminishes sexual satisfaction, and adversely influences subjective health perception. Given these multifaceted impacts, effective management and preventive strategies for recurrent vulvovaginal disorders remain critical areas of clinical research [6].

Vaginal health plays a fundamental role in the overall well-being of women. It is essential for sexual function, preventing infections, and avoiding discomfort such as dryness or pain, and is closely linked to the integrity of the vaginal microbiome, which is primarily dominated by *Lactobacillus* species [7,8]. These bacteria maintain an optimal low pH < 4.5 by metabolizing glycogen to lactic acid, creating an environment inhibiting pathogenic growth and supporting a balanced microbiota [3]. A significant reduction or depletion of *Lactobacillus* species can lead to vaginal dysbiosis, often characterized by anaerobic bacterial and fungal overgrowth. This imbalance is linked to conditions such as BV, VVC, sexually transmitted infections (STIs), and adverse pregnancy outcomes [9].

Maintaining a Lactobacillus-dominated vaginal microbiota appears to play an essential role in preserving vulvovaginal homeostasis. Lactobacillus species are hypothesized to contribute significantly to vaginal health by generating lactic acid, thereby maintaining an acidic vaginal environment (pH < 4.5), and potentially producing antimicrobial agents such as hydrogen peroxide and bacteriocins. Furthermore, these bacteria may facilitate local immune modulation and epithelial barrier integrity by adhering to vaginal epithelial cells, thereby limiting colonization by potential pathogens [10,11]. Alterations towards a Lactobacillus-deficient microbiome characterized by diverse anaerobic bacteria appear to be associated with increased vulnerability to vaginal infections and adverse outcomes, including preterm birth [12]. Personalized therapeutic strategies that aim to re-establish a physiologically beneficial Lactobacillus-rich microbiota may be crucial in optimizing long-term therapeutic outcomes.

The treatment of symptoms should be tailored to the underlying cause; however, international guidelines consistently recommend vaginal preparations over oral therapies for both infectious and non-infectious vulvovaginitis [13,14]. Vaginal preparations offer localized drug delivery, resulting in high concentrations at the site of infection, rapid symptom relief, and a reduced risk of systemic side effects [15,16]. In contrast, oral therapies are associated with a higher incidence of systemic adverse effects. Nevertheless, the importance of health-supporting vaginal preparations is undeniable, both as complementary elements of therapy and in restoring normal flora post-treatment, as well as in preventing recurrences [17].

In a previous study, we evaluated the therapeutic potential of a zinc containing hydroxyethyl cellulose (HEC)-based hydrogel for managing GSM, demonstrating its suitability as a first-line treatment in alignment with international guidelines [13]. The study concluded that the zinc-containing vaginal hydrogel is an effective, topical, and nonhormonal treatment for GSM, providing significant symptom relief. The gel’s formulation, incorporating hydroxyethyl cellulose, lactic acid, and zinc sulfate, contributes to its ability to enhance vaginal hydration and maintain an acidic pH conducive to vaginal health [18]. Hydrogels possess a three-dimensional crosslinked molecular structure that enables them to absorb substantial amounts of water and expand within a physiological environment [19]. Hydrogels exhibit physical properties resembling living tissues because of their high water content, elastic consistency, and low interfacial tension with biological fluids and water [20]. This characteristic, which replicates natural tissues, makes hydrogels highly biocompatible and has attracted considerable attention in biomaterial applications over the past several decades [19].

The convergence of advancements in biomaterial science and microbiome research is the opening novel possibilities for the management of vulvovaginal disorders. The zinc-containing hydrogel represents a potentially multifunctional therapeutic intervention, which could provide symptomatic relief through improved lubrication and support of optimal vaginal pH. Furthermore, zinc may contribute beneficial antimicrobial and immunomodulatory properties, potentially enhancing local immune responses. Its preliminary success in managing genitourinary syndrome of menopause (GSM), along with promising early findings regarding its role in infection prophylaxis, suggests the possibility that it might be effective in addressing a broader spectrum of vulvovaginal symptoms. Given these encouraging findings, further research is warranted to fully elucidate the clinical utility and mechanisms of action of zinc-containing hydrogels as non-hormonal treatments for vulvovaginal health [21].

We hypothesize that the zinc-containing hydrogel, previously shown to be effective in alleviating the symptoms of GSM, may also be therapeutically effective in alleviating the vulvovaginal symptoms caused by other etiologies [18].

## 2. Results and Discussion

### 2.1. Results

**Figure 1:**
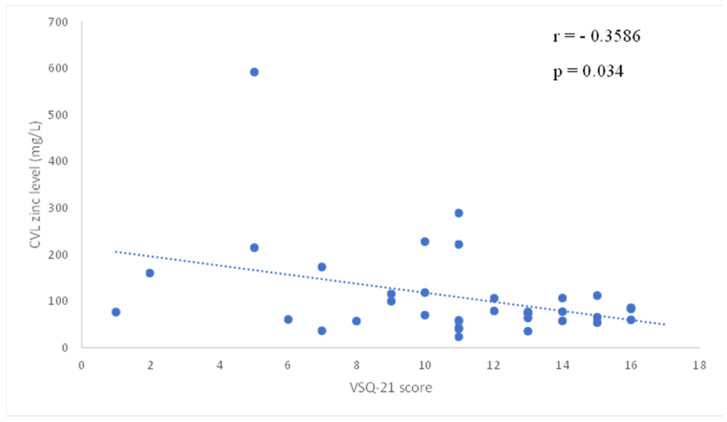
Correlation of CVL zinc levels and VSQ21 scores at enrollment. Data demonstrate a significant inverse correlation between baseline CVL zinc levels and VSQ-21 scores, meaning that higher zinc levels were associated with fewer vulvovaginal symptoms.

**Figure 2:**
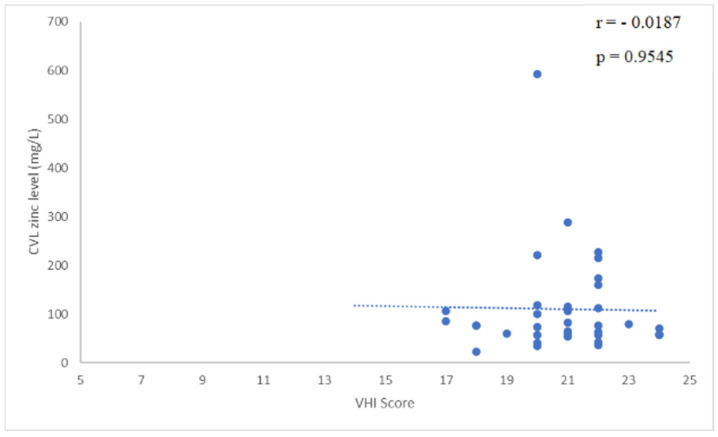
Correlation of CVL zinc levels and VHI scores at enrollment. Data shows no significant correlation between baseline CVL zinc levels and VHI scores, indicating that while zinc levels may influence subjective symptoms as seen in VSQ-21, their direct impact on objective vaginal health measures remains unclear. The minimum VHI score is 5, which is why the *x*-axis starts at this value.

The study enrolled 37 women with a mean age of 37 ± 12 years. The majority of participants were premenopausal, with a median parity of 1 (range: 0–3) and a mean body mass index of 26.6 ± 5.3 kg/m^2^. Table 1 shows the demographics and clinical characteristics of participants. All participants completed the 12-week study period, and no adverse events were reported. The VHI scores showed a statistically significant improvement from baseline to week 12, increasing from 20.78 ± 1.74 at baseline to 23.64 ± 2.59 (*p* < 0.01). The VSQ-21 scores decreased significantly, reflecting an alleviation of symptoms. The baseline score of 10.78 ± 3.66 dropped to 3.17 ± 4.16 by week 12 (*p* < 0.01). Participants reported marked reductions in itching, burning, and discharge severity. Vaginal pH also showed a significant reduction, improving from 4.03 ± 0.42 at baseline to 3.71 ± 0.48 at week 12 (*p* < 0.01), indicating a healthier vaginal environment (Table 2). The mean baseline zinc concentration in cervicovaginal lavage (CVL) samples was 110 ± 102 µg/L. By week 4, CVL zinc levels measured 62 ± 48 µg/L, representing a statistically significant change from baseline (*p* < 0.01). At week 8, zinc levels measured 80 ± 55 µg/L, maintaining statistical significance compared to baseline (*p* = 0.04). By week 12, zinc levels reached 99 ± 92 µg/L, and this difference was no longer statistically significant (NS), as shown in Table 3. Analysis of the correlation between CVL zinc levels and clinical parameters revealed an inverse relationship with VSQ21 scores at enrollment (r = −0.3586, *p* = 0.034). In contrast, no significant correlation was found between CVL zinc levels and VHI scores at enrollment (r = −0.0187, *p* = 0.9545), as shown in Figure 1 and Figure 2. Participants expressed high satisfaction with the gel, noting its ease of use and perceived effectiveness. Many reported an improvement in overall vaginal comfort and reduced recurrence of symptoms. The adherence rate was over 95%, and all participants followed the recommended application schedule.

### 2.2. Discussion

To our knowledge, this study is the first to demonstrate that a zinc-containing vaginal hydrogel significantly improves vaginal health in women with a variety of vulvovaginal symptoms. Our findings indicate that this novel therapeutic approach significantly alleviates symptoms such as vaginal dryness, itching, and dyspareunia. The VSQ-21 scores improved significantly post-treatment, underscoring the potential of zinc-based formulations as a non-hormonal, drug-free, and locally acting alternative treatment for improving vulvovaginal symptoms. Vulvovaginal symptoms profoundly impact a woman’s quality of life. These symptoms are often associated with anxiety, depression, and reduced self-esteem [22]. An imbalance in the vaginal microbiota can lead to recurring infections, which can be challenging to treat. Studies highlight that maintaining a healthy vaginal microbiota dominated by *Lactobacillus* species is crucial for preventing infections and promoting overall reproductive health [23,24]. Given the profound impact of these conditions, there is an urgent need for innovative and effective interventions, such as the zinc-containing hydrogel, that targets both symptom relief and microbiota restoration.

Hydrogels are hydrophilic materials featuring a three-dimensional crosslinked molecular structure, enabling them to retain large quantities of water without dissolving in physiological conditions, and have extensive medical applications, including use in vaginal drug delivery systems, burn treatment, and wound care [25,26]. Hydrogels are optimal formulations for vaginal applications due to their superior mucoadhesive properties, excellent compatibility with biological tissues, and high water absorption capacity. These attributes make hydrogels highly effective in maintaining vaginal hydration and ensuring the efficient delivery of active pharmaceutical ingredients [20,27]. Another important benefit of hydrogels is their high comfort level for patients. Unlike creams and ointments, often perceived as messy or greasy, hydrogels have a non-greasy, lightweight texture that enhances patient satisfaction and adherence [27]. In clinical evaluations, more than 94% of participants reported hydrogels to be comfortable and satisfactory, highlighting their potential to address common barriers to adherence observed with other formulations [18].

Biocompatibility represents another critical advantage of hydrogels, particularly those formulated with cellulose derivatives such as hydroxyethyl cellulose (HEC). These hydrogels, derived from semi-synthetic cellulose polymers, are inherently less likely to cause irritation or allergic reactions compared to fully synthetic polymers like carbomer [28]. Additionally, their high water content not only provides optimal hydration to the vaginal mucosa but also supports epithelial repair and barrier function. Notably, the maintenance of vaginal pH within the physiologically acidic range ensures a favorable environment for *Lactobacillus* dominance, which is essential for protecting against infections such as bacterial vaginosis and candidiasis [29]. Due to their hydrophilic nature, hydrogels do not disrupt this delicate pH balance, further underscoring their clinical utility [30].

Zinc-containing vaginal formulations have significant potential in maintaining vaginal health, particularly by supporting *Lactobacillus* species, which are the dominant elements of the vaginal microbiota. Zinc has been shown to positively affect specific *Lactobacillus* species, including *Lactobacillus plantarum* and *Lactobacillus acidophilus*, by promoting their growth. Research indicates that zinc plays a fundamental role in the metabolism and physiology of these bacteria, influencing key cellular processes. Zinc-deficient environments have been observed to prolong the bacterial lag phase, while optimal zinc concentrations significantly reduce this delay, resulting in accelerated bacterial proliferation. These findings underscore zinc’s critical contribution to *Lactobacillus* species’ survival and growth dynamic [31]. Zinc can help maintain the normal acidic pH of the vagina, thereby inhibiting pathogenic bacteria while creating favorable conditions for beneficial microbes. These findings confirm that zinc-containing formulations may not only alleviate symptoms but also support the long-term health of vaginal tissues [17]. Previous research and the literature have established the critical role of vaginal pH in maintaining vaginal health [23].

Building on this understanding of the role of zinc in vaginal health, we used the CVL method to assess its presence in the vaginal environment following the use of a zinc-containing hydrogel. CVL zinc levels measured at week 12 were not significantly different from baseline, despite the observed improvements in vulvovaginal symptoms. This finding suggests that the zinc from the hydrogel is efficiently absorbed and becomes readily available to the vaginal epithelium, where it can exert its effects. This may be due to the fact that zinc from the HEC-based hydrogel has been well absorbed, which could be beneficial, as our previous research indicates that oral supplementation might not achieve sufficient absorption to exert an optimal effect on the vaginal epithelium [32].

The correlation analysis revealed an inverse relationship between CVL zinc levels and VSQ21 scores at enrollment (r = −0.3586, *p* = 0.034). This finding aligns with prior research indicating that zinc plays a role in maintaining vaginal mucosal integrity and supporting epithelial barrier function, which may contribute to symptom alleviation [24,31,32]. As the VSQ21 questionnaire encompasses various vulvovaginal symptoms, including irritation, dryness, and discomfort, the observed association underscores zinc’s potential role in enhancing vaginal health. In contrast, no significant correlation was observed between CVL zinc levels and VHI scores at enrollment (r = −0.0187, *p* = 0.9545). VHI serves as an objective, clinician-administered assessment of vaginal health, whereas VSQ-21 captures patients’ subjective symptomatology. As symptom perception varies among individuals, the significant improvements in VSQ-21 scores provide compelling evidence of the hydrogel’s positive impact on patients’ quality of life.

We observed that the vaginal pH after 4, 8, and 12 weeks was significantly lower than the pH of the gel itself (4.4). This finding suggests that the observed effect cannot be attributed solely to the gel’s acidic pH. Instead, it is likely the result of a synergistic interaction between the acidic environment and the zinc-containing gel, which together contribute to the maintenance of an optimal vaginal pH and overall health.

The study demonstrates several strengths. One of its key strengths lies in its clinical relevance, as it addresses a significant gap in managing vulvovaginal symptoms, providing an alternative treatment for women. The zinc-containing hydrogel has proven effective in symptom relief and vaginal health improvement, underscoring its potential as a valuable therapeutic option. Additionally, the research employs validated outcome measures, such as the VSQ-21 and VHI. Another strength is the hydrogel’s safety profile and high adherence rates, with over 95% of participants following the application protocol and reporting minimal adverse effects, highlighting the practicality and patient acceptability of the treatment. Furthermore, the innovative formulation of the hydrogel, which combines hydroxyethyl cellulose, lactic acid, and zinc sulfate, provides a multifaceted approach to improving vaginal health by enhancing hydration, maintaining an optimal pH, and supporting a balanced microbiota.

This study also has some limitations. The small sample size of 37 participants limits the statistical power and generalizability of the findings, emphasizing the need for larger studies to confirm the results. The short follow-up period of 12 weeks does not allow for an assessment of the long-term effects of the treatment, such as sustained symptom relief or its impact on recurrent vulvovaginal conditions. In addition, women with a variety of different vulvovaginal symptoms were enrolled, rather than women with only one clinical diagnosis. The lack of a placebo-controlled design further restricts the ability to draw definitive conclusions about the specific effects of the active components. Placebo-controlled studies are necessary; however, their implementation presents a significant challenge, as most placebo gels are inherently acidic, and introducing a non-acidic gel into the vaginal environment would raise ethical concerns. Finally, the study population consists of women from a single geographic and clinical setting, which may limit the applicability of the findings to more diverse populations with different demographic, cultural, and health profiles. These limitations underscore the need for future research to build on the promising results of this study.

## 3. Conclusions

This study demonstrates that the zinc-containing hydroxyethyl cellulose-based vaginal hydrogel significantly improves vaginal health by alleviating different vulvovaginal symptoms. Its formulation provides a safe and patient-friendly alternative to oral therapies with no systemic side effects. The gel addresses symptomatic relief and long-term microbiota support by potentially enhancing *Lactobacillus* growth and maintaining an optimal acidic pH. These findings suggest that it could serve as a potential first-line treatment or as a complementary element of therapy for a variety of vulvovaginal symptoms.

## 4. Materials and Methods

A prospective cohort study was performed with the enrollment of thirty-seven women with different vulvovaginal symptoms (itching, burning, irritation, pain, dryness, discharge, and odor) at the outpatient clinic of the Department of Obstetrics and Gynecology, University of Debrecen, Hungary, between April 2023 and April 2024. This clinical trial was conducted to evaluate the efficacy of a commercially available zinc-containing vaginal hydrogel (Juvia vaginal gel; Fempharma, LLC., Debrecen, Hungary) in improving vaginal health in women with different vulvovaginal symptoms. The key ingredients of the gel are water, hydroxyethyl cellulose, zinc sulfate heptahydrate at a concentration of 20 mM, and lactic acid. The study was approved by the Hungarian National Institutional Review Medical Research Council (approval number 3282-7/2023/EUIG), and all participants provided written informed consent. We enrolled women aged 18 years or older presenting with a variety of vulvovaginal symptoms, including vaginal discharge, odor, itching, pain, dysuria, skin irritation, burning, and dyspareunia. Exclusion criteria included pregnancy, breastfeeding, abnormal cervical cytology within the last year, recent use of antifungal medications, known allergies to zinc or azoles, and liver disease. Patients with sexually transmitted diseases like trichomonas, chlamydia, or gonorrhea were also excluded. Participants underwent clinical evaluations at four visits: baseline (week 0), week 4, week 8, and week 12.

At the initial visit, the baseline diagnosis was established and participants received oral antifungal treatment if a vaginal yeast infection was present. Patients with other vulvovaginal conditions or infection did not receive any other treatment unless there was an obvious reason present. Women were asked to use the vaginal gel daily for 2 weeks and twice per week thereafter to complete the 12 weeks study period. The research team recorded demographic and clinical data at baseline, including age, body mass index, parity, and history of recurrent infections. The following assessments were performed at each visit: gynecological examination using a plastic speculum, which does not contain zinc or copper, to assess clinical signs of infection, vaginal discharge, and irritation. Following the assessment of symptoms, a CVL sample was collected for zinc measurement. CVL was collected by lavaging the cervix and vaginal walls with 10 mL of normal sterile saline and then aspirating the pooled fluid with a plastic syringe. The lavage fluid was introduced into the vagina with a plastic syringe for 60 s with three successive aspirations and emptying to the vaginal walls and cervix. The total amount of wash fluid was recovered from the posterior fornix by syringe aspiration. CVL samples were collected and stored at −80 °C until analysis. The process of preparing the CVL sample was as follows: a volume of 3.00 mL of each liquid sample was measured using an automatic pipette, followed by the evaporation of water content on an electric heating plate. Subsequently, the samples were digested with a mixture of 3 mL of 65% HNO_3_ (Sigma Aldrich, Merck, Budapest, Hungary) and 1 mL of 30% H_2_O_2_ (VWR). The digested solutions were quantitatively transferred into polypropylene (PP) centrifuge tubes, diluted to a final volume of 12 mL with ultrapure water (Synergy UV Millipore), and filtered through a 0.45 µm PVDF membrane filter. The measurement protocol for the CVL sample was then conducted. The elemental composition of the samples was determined using an ICP-OES 5110 Vertical Dual View instrument (Agilent Technologies, Santa Clara, CA, USA). The analysis was conducted with an Agilent SPS4 automated sample injector, a Meinhard^®^ nebulizer, and a double-pass spray chamber. A seven-point matrix-matched calibration series was employed, prepared from a 1000 mg/L spectroscopic standard stock solution (ICP IV, Certipur, Merck) and analytical-grade sodium chloride (Molar Chemicals, Halásztelek, Hungary) using 0.1 M HNO_3_. The measurement accuracy was verified through matrix-matched independent standard recovery testing, with an acceptance criterion of ±5% recovery. The operating parameters of ICP-OES are shown in Table 4.

We used validated questionnaires to assess the severity of symptoms. The severity of vulvovaginitis symptoms, including vaginal discharge, odor, itching, pain, dysuria, skin irritation, burning, and dyspareunia, was recorded using the VSQ-21. The VSQ-21 is a validated tool where higher scores indicate more bothersome symptoms [33]. VHI is a clinical evaluation method to assess vaginal health by scoring: elasticity, fluid secretion, pH, epithelial mucosa (integrity), and moisture components. Each component is scored on a scale of 1 (worst) to 5 (best). Higher scores indicate healthier vaginal conditions [34]. During the first clinical visit, we instructed the participants to self-administer 2 mL of the gel intravaginally with a provided applicator for 12 weeks: daily for the first 2 weeks and twice weekly for the remaining 10 weeks. We monitored adherence to the intervention using participant logs and documented adverse events at each visit. The primary outcome was the reduction in VSQ scores. Secondary outcomes included changes in VHI and Vaginal pH scores.

Sample size calculation was performed using G*Power 3.1.9.7 software with α = 5% and β = 90%. To detect a mean increase of 3 points in the VHI score (15% improvement) from the baseline VHI score of 20 ± 2.5, a sample size of 32 participants was required. Accounting for a 15% dropout rate, 37 participants were planned for enrollment. Statistical analyses were conducted using SPSS 29 software (Systat Software Inc., San Jose, CA, USA). Continuous variables were expressed as means ± standard deviation (SD) and compared using paired *t*-tests for within-group comparisons. Pearson’s correlation analysis was performed to assess the relationship between continuous variables, with correlation coefficients (r-values) reported to quantify the strength and direction of associations. Statistical significance was set at *p* < 0.05.

## Figures and Tables

**Table 1:** Demographics and clinical characteristics. The data are presented as number or mean ± standard deviation. Gravida: number of pregnancies. Parity: number of deliveries. BMI: mean body mass index.

	(N = 37)
Age (years, mean ± SD)	37 ± 12
Gravida (median, range)	1 (0–4)
Parity (median, range)	1 (0–3)
BMI (kg/m^2^, mean ± SD)	26.6 ± 5.3
Postmenopausal (n, %)	5 (14)

**Table 2:** Changes in vaginal health parameters during treatment. The data are presented as mean ± standard deviation. Lower vaginal pH indicates a healthier vaginal microbiome. An increase in the VHI score signifies an improvement in vaginal health. A decrease in the VSQ score represents an improvement in symptoms and quality of life.

	Baseline	Week 4	*p*-Value (Baseline vs. W4)	Week 8	*p*-Value (Baseline vs. W8)	Week 12	*p*-Value (Baseline vs. W12)
Vaginal pH	4.03 ± 0.42	3.91 ± 0.26	0.12	3.71 ± 0.22	<0.01	3.71 ± 0.48	<0.01
Vaginal Health Index (VHI)	20.78 ± 1.74	23.27 ± 2.09	<0.01	23.88 ± 1.98	<0.01	23.64 ± 2.59	<0.01
Vulvovaginal Symptom Questionnaire-21 (VSQ-21)	10.78 ± 3.66	5.24 ± 4.78	<0.01	3.00 ± 4.00	<0.01	3.17 ± 4.16	<0.01

**Table 3:** Changes in CVL zinc levels over time. Data shows how CVL zinc levels change over time, initially decreasing significantly from baseline to week 4, then gradually increasing by week 8 and stabilizing by week 12. This pattern suggests that zinc from the hydrogel is absorbed and utilized in the vaginal environment, with levels adjusting dynamically over time.

	Baseline	Week 4	*p*-Value (Baseline vs. W4)	Week 8	*p*-Value (Baseline vs. W8)	Week 12	*p*-Value (Baseline vs. W12)
Cervicovaginal lavage zinc level (µg/L, mean ± SD)	110 ± 102	62 ± 48	<0.01	80 ± 55	0.04	99 ± 92	NS

**Table 4:** The operating parameters of ICP-OES.

Parameter	Value
Pump speed (rpm)	15
Sample uptake time (sec)	15
Rinse time (sec)	30
Stabilization time (sec)	20
Nebulizer gas flow (L/min)	0.70
Number of repetitions	3
Wavelength (nm)	213,857
Observation Mode	axial
Reading Time (sec)	10

## Data Availability

The original contributions presented in this study are included in the article. Further inquiries can be directed to the corresponding authors.

## References

[1] Sobel J.D. (1990). Vaginal infections in adult women. Med. Clin. N. Am..

[2] Powell A.M., Nyirjesy P. (2014). Recurrent vulvovaginitis. Best Pract. Res. Clin. Obstet. Gynaecol..

[3] Itriyeva K. (2020). Evaluation of vulvovaginitis in the adolescent patient. Curr. Probl. Pediatr. Adolesc. Health Care.

[4] Kingsberg S.A., Wysocki S., Magnus L., Krychman M.L. (2013). Vulvar and Vaginal Atrophy in Postmenopausal Women: Findings from the REVIVE (REal Women’s VIews of Treatment Options for Menopausal Vaginal ChangEs) Survey. J. Sex. Med..

[5] Lev-Sagie A., Amsalem H., Gutman Y., Esh-Broder E., Daum H. (2020). Prevalence and Characteristics of Postpartum Vulvovaginal Atrophy and Lack of Association With Postpartum Dyspareunia. J. Low. Genit. Tract Dis..

[6] Fukazawa E.I., Witkin S.S., Robial R., Vinagre J.G., Baracat E.C., Linhares I.M. (2019). Influence of recurrent vulvovaginal candidiasis on quality of life issues. Arch. Gynecol. Obstet..

[7] Graziottin A. (2024). Maintaining vulvar, vaginal and perineal health: Clinical considerations. Womens Health.

[8] Redondo-Lopez V., Cook R.L., Sobel J.D. (1990). Emerging Role of Lactobacilli in the Control and Maintenance of the Vaginal Bacterial Microflora. Rev. Infect. Dis..

[9] Zheng N., Guo R., Wang J., Zhou W., Ling Z. (2021). Contribution of Lactobacillus iners to Vaginal Health and Diseases: A Systematic Review. Front. Cell. Infect. Microbiol..

[10] Petrova M.I., van den Broek M., Balzarini J., Vanderleyden J., Lebeer S. (2013). Vaginal microbiota and its role in HIV transmission and infection. FEMS Microbiol. Rev..

[11] Lebeer S., Vanderleyden J., De Keersmaecker S.C.J. (2010). Host interactions of probiotic bacterial surface molecules: Comparison with commensals and pathogens. Nat. Rev. Microbiol..

[12] Gudnadottir U., Debelius J.W., Du J., Hugerth L.W., Danielsson H., Schuppe-Koistinen I., Fransson E., Brusselaers N. (2022). The vaginal microbiome and the risk of preterm birth: A systematic review and network meta-analysis. Sci. Rep..

[13] Kaunitz A.M., Manson J.E. (2014). ACOG Practice Bulletin No. 141: Management of menopausal symptoms. Obstet. Gynecol..

[14] Pappas P.G., Kauffman C.A., Andes D.R., Clancy C.J., Marr K.A., Ostrosky-Zeichner L., Reboli A.C., Schuster M.G., Vazquez J.A., Walsh T.J. (2016). Clinical Practice Guideline for the Management of Candidiasis: 2016 Update by the Infectious Diseases Society of America. Clin. Infect. Dis..

[15] Sobel J.D., Ferris D., Schwebke J., Nyirjesy P., Wiesenfeld H.C., Peipert J., Soper D., Ohmit S.E., Hillier S.L. (2006). Suppressive antibacterial therapy with 0.75% metronidazole vaginal gel to prevent recurrent bacterial vaginosis. Am. J. Obstet. Gynecol..

[16] Bassi A., Sharma G., Deol P.K., Madempudi R.S., Kaur I.P. (2023). Preclinical Potential of Probiotic-Loaded Novel Gelatin–Oil Vaginal Suppositories: Efficacy, Stability, and Safety Studies. Gels.

[17] Mollazadeh-Narestan Z., Yavarikia P., Homayouni-Rad A., Samadi Kafil H., Mohammad-Alizadeh-Charandabi S., Gholizadeh P., Mirghafourvand M. (2023). Comparing the Effect of Probiotic and Fluconazole on Treatment and Recurrence of Vulvovaginal Candidiasis: A Triple-Blinded Randomized Controlled Trial. Probiotics Antimicrob. Proteins.

[18] Takacs P., Kozma B., Erdodi B., Jakab A., Larson K., Poka R. (2019). Zinc-containing Vaginal Moisturizer Gel Improves Postmenopausal Vulvovaginal Symptoms: A Pilot Study. J. Menopausal Med..

[19] Jabbari E. (2018). Hydrogels for Cell Delivery. Gels.

[20] Hamidi M., Azadi A., Rafiei P. (2008). Hydrogel nanoparticles in drug delivery. Adv. Drug Deliv. Rev..

[21] Roselletti E., Pericolini E., Nore A., Takacs P., Kozma B., Sala A., De Seta F., Usher M.C.J., Brown G.D., Wilson D. (2023). Zinc prevents vaginal candidiasis by inhibiting expression of an inflammatory fungal protein. Sci. Transl. Med..

[22] Shroff S. (2023). Infectious Vaginitis, Cervicitis, and Pelvic Inflammatory Disease. Med. Clin. N. Am..

[23] Chen R., Li R., Qing W., Zhang Y., Zhou Z., Hou Y., Shi Y., Zhou H., Chen M. (2022). Probiotics are a good choice for the treatment of bacterial vaginosis: A meta-analysis of randomized controlled trial. Reprod. Health.

[24] Zhu B., Tao Z., Edupuganti L., Serrano M.G., Buck G.A. (2022). Roles of the Microbiota of the Female Reproductive Tract in Gynecological and Reproductive Health. Microbiol. Mol. Biol. Rev..

[25] Surowiecka A., Strużyna J., Winiarska A., Korzeniowski T. (2022). Hydrogels in Burn Wound Management—A Review. Gels.

[26] Blinov A.V., Kachanov M.D., Gvozdenko A.A., Nagdalian A.A., Blinova A.A., Rekhman Z.A., Golik A.B., Vakalov D.S., Maglakelidze D.G., Nagepetova A.G. (2023). Synthesis and Characterization of Zinc Oxide Nanoparticles Stabilized with Biopolymers for Application in Wound-Healing Mixed Gels. Gels.

[27] Rüther L., Voss W. (2021). Hydrogel or ointment? Comparison of five different galenics regarding tissue breathability and transepidermal water loss. Heliyon.

[28] Takacs P., Kozma B., Rátonyi D., Kozma B., Attila K., Fenyvesi F., Sipos A.G. (2024). Development and Bioavailability Assessment of an Estriol-Containing Vaginal Hydrogel. Gels.

[29] Dos Santos A.M., Carvalho S.G., Araujo V.H.S., Carvalho G.C., Gremião M.P.D., Chorilli M. (2020). Recent advances in hydrogels as strategy for drug delivery intended to vaginal infections. Int. J. Pharm..

[30] Livne S., Simantov S., Rahmanov A., Jeffet U., Sterer N. (2022). Hydroxyethyl Cellulose Promotes the Mucin Retention of Herbal Extracts Active against Streptococcus mutans. Materials.

[31] Huynh U., Qiao M., King J., Trinh B., Valdez J., Haq M., Zastrow M.L. (2022). Differential Effects of Transition Metals on Growth and Metal Uptake for Two Distinct Lactobacillus Species. Microbiol. Spectr..

[32] Takacs P., Damjanovich P., Sipos A.G., Kozma B. (2020). The effect of oral zinc supplementation on cervicovaginal lavage fluid zinc level. Eur. J. Obstet. Gynecol. Reprod. Biol..

[33] Erekson E.A., Yip S.O., Wedderburn T.S., Martin D.K., Li F., Choi J.N., Kenton K.S., Fried T.R. (2013). The Vulvovaginal Symptoms Questionnaire: A questionnaire for measuring vulvovaginal symptoms in postmenopausal women. Menopause.

[34] Mehta A., Bachmann G. (2008). Vulvovaginal complaints. Clin. Obstet. Gynecol..

